# A functional polymorphism of *SSBP1* gene predicts prognosis and response to chemotherapy in resected gastric cancer patients

**DOI:** 10.18632/oncotarget.22864

**Published:** 2017-12-02

**Authors:** Qiuchen Li, Falin Qu, Renli Li, Xianli He, Yulong Zhai, Weigang Chen, Yong Zheng

**Affiliations:** ^1^ Department of Gastroenterology, First Affiliated Hospital of the Medical College, Shihezi University, Shihezi, Xinjiang, 832008, China; ^2^ Department of General Surgery, Tangdu Hospital, The Fourth Military Medical University, Xi’an, Shaanxi, 710038, China; ^3^ Department of General Surgery, The Fourth Hospital of Chinese PLA, Xining, Qinghai, 810007, China

**Keywords:** single nucleotide polymorphism, gastric cancer, prognosis, single-strand DNA-binding protein 1, chemotherapy

## Abstract

Growing evidence has indicated that single-stranded DNA-binding proteins 1 (*SSBP1*) is involved in tumor initiation and progression. However, effects of single nucleotide polymorphisms (SNPs) in *SSBP1* gene on gastric cancer (GC) prognosis are still unknown. In present study, two functional SNPs from *SSBP1* were selected and genotyped in a large cohorts of 1030 resected GC patients (326 in the training set, 704 in the validation set) to explore the association of SNPs with patients’ survival. The rs6976500 G allele (CG/GG) genotypes were found significantly associated with both worse overall survival (OS) and recurrence-free survival (RFS) in the training and the independent validation set when compared to C allele genotype, which reaching a more robust statistical significance in the pooled analysis. Furthermore, integration of rs6976500 genotypes and TNM stage significantly improved the prognosis prediction models based on TNM stage alone. In addition, only carriers with at least one G allele of rs6976500 gained significant survival benefit from FOLFOX-based ACT. Mechanistically, SNP rs6976500 G allele genotype could significantly decrease promoter transcriptional activity and markedly reduce expression level of *SSBP1* compared with the C allele genotype in GC cells. This was further substantiated by immunohistochemical assay in 70 GC tissue samples. Our study presents the first evidence that SNP rs6976500 G allele genotypes might contribute to GC prognosis by attenuating *SSBP1* promoter activity and gene expression, and provides the guidance in refining therapeutic decisions of GC patients. Further exploration on its function is needed to clarify the exact biological mechanism behind.

## INTRODUCTION

Gastric cancer (GC) is globally the fourth most common cancer and the second leading cause of cancer death, accounting for approximately 8% of the total cancer cases and 10% of total cancer-related deaths worldwide [1]. Almost 70% of GC cases occurred in developing nations, and half of them occurred in East Asia (prominently in China) [2]. Despite decades of efforts, the prognosis of GC patients remains dismal, with 5-year survivals below 24% [3]. Clinically, the Tumor, Node, Metastasis (TNM) staging system is recommended as the gold standard for therapeutic decision making and prognostic prediction of GC patients. However, due in part to tumor heterogeneity, patients with the same TNM stage often exhibit distinct clinical outcomes [4]. More importantly, although the TNM staging system divides patients into subgroups with different clinical outcomes, but it provides little information about treatment effect in individual patients. Therefore, it is imperative to discovery novel biomarkers for GC patients to complement the TNM staging system to improve the prediction of prognosis and guide treatment decision.

GC is generally recognized as disease mainly affected by environmental exposures and carcinogens in the diet [5]. However, in the last two decades, a growing number of researches have suggested that GC pathogenesis also involves host genetic factors [6]. Therefore, searching for susceptibility genes contributing to GC initiation and development is currently under intense investigation. More recently, a line of evidence has suggested that germline variation such as single nucleotide polymorphisms (SNPs) can not only affect GC susceptibility [7], but also confer GC patients with different prognosis [8–10], which further confirmed that genetic background play an essential role in gastric carcinogenesis and progression.

Mitochondrial single-strand DNA-binding protein 1 (SSBP1) is a housekeeping gene involved in mitochondrial biogenesis by maintaining the mitochondrial genome stability [11]. As a subunit of the SSB complex, SSBP1 can regulate several important cellular physiological activities, such as maintenance of mitochondrial DNA content [12] and changes in metabolic status [13]. Recently, aberrant expression of *SSBP1* has been frequently reported in many cancers [14–16], suggesting a possible functional link between *SSBP1* and human cancer. The subsequent mechanism research demonstrated that *SSBP1* may act as a tumor suppressor to control tumorigenesis and progression [15, 17], and decreased expression of *SSBP1* significantly increased the sensitivity to ionizing radiation in lung cancer [18]. These researches have suggested that SSBP1 is involved in tumor initiation and progression and cellular injury response by regulation of mitochondrial function and cell metabolism. Therefore, SSBP1 are regarded as a promising prognostic marker and an therapeutic target for human cancer. However, as far as we are aware, there is currently no literature reporting *SSBP1* gene associated with GC. In view of the crucial roles of SSBP1 in genome stability and tumorigenesis, theoretically, functional genetic variants in *SSBP1* gene, which potentially influence *SSBP1* expression, might facilitate the process of GC development.

In the present study, we firstly evaluated the effects of two potential functional SNPs in the 5′-untranslated region (UTR) of *SSBP1* on GC patients’ prognosis in a training cohort (*n* = 326) and found that SNP rs6976500 contributed to poor survival of GC, which further validated in an independent validation cohort (*n* = 704). Furthermore, functional assays were performed to explore the effect of SNP rs6976500 on the regulation of *SSBP1* gene expression by using luciferase assays. Our finding indicates that genetic variants in *SSBP1* gene contribute to GC prognosis by altering SSBP1 promoter activity and gene expression, which is an important molecular mechanism of GC progression.

## RESULTS

### Patient characteristics and prognosis analysis

Demographic and clinical characteristics of GC patients were summarized in Supplementary Table 1. Due to the late enrollment due dates of GC patients for the independent validation cohort in this ongoing molecular epidemiological study, the median follow-up time in the validation cohort was significantly shorter than that in the training cohort (58 months *vs*. 76 months). Thus patients in the training cohort had much higher rates of recurrence (76.1%) and death (63.8%) than those in the validation cohort (68.8% and 55.4%, respectively) (both *P* < 0.05). During the follow-up period, 732 patients (248 and 484 in the training and validation cohort, respectively) developed recurrence and 598 patients (208 and 390 in the training and validation cohort, respectively) died of GC. Most patients (66.3%) received adjuvant chemotherapy (ACT) after surgery. Only 11 patients received postoperative radiotherapy plus ACT. In light of the small number of patients, we neglected the analysis of radiotherapy. Among the 683 patients receiving ACT, 665 (97.3%) received the FOLFOX regimen. In addition, of the 89 patients diagnosed in stage IV, 57 developed liver metastasis, 14 had spread to enterocoelia, and 18 had spread to other organs such as ovary, oviduct, vagina and lung. No significant differences were found between training cohort and validation cohort with respect to host characteristics, such as age, tumor site, tumor size, TNM stage, Lauren classification, differentiation and ACT (*P* value ranging from 0.079 to 0.898).

We further conducted a multivariate analysis to evaluate the prognostic effects of all selected host characteristics on OS and RFS using Cox regression model. As shown in Table 1, the risk of death or recurrence for GC was progressively increased from stage I to stage IV among training cohort, validation cohort and pooled analysis (*P* for trend < 0.001, for all). Patients with diffuse type or poor differentiated tumor exhibited markedly worse OS and RFS than those with intestinal type or well/moderate differentiated tumor in all patient cohorts (*P* < 0.05, for all). In addition, patients who received FOLFOX-based ACT after surgery had a significantly decreased risks of recurrence and death in training cohort (HR = 0.79 and 0.53, respectively), validation cohort (HR = 0.58 and 0.68, respectively) and pooled analysis (HR = 0.68 and 0.63, respectively) when compared with those who were treated by surgery alone.

**Table 1:** Distribution of patients’ characteristics and prognosis analysis in the training set and the validation set

Variables	Training set (*n* = 326)				Validation set (*n* = 704)				Pooled analysis (*n* = 1030)			
	Deaths/Total 208/326	HR^a^ (95% CI)	Relapse/Total 248/326	HR^a^ (95% CI)	Deaths/Total 390/704	HR^a^ (95% CI)	Relapse/Total 484/704	HR^a^ (95% CI)	Deaths/Total 598/1030	HR^a^ (95% CI)	Relapse/Total 732/1030	HR^a^ (95% CI)
Age												
≤57	102/160	Reference	119/160	Reference	181/339	Reference	225/339	Reference	283/499	Reference	344/499	Reference
>57	106/166	0.92 ((0.79–1.28)	129/166	0.90 (0.80–1.27)	209/365	1.04 (0.85–1.27)	259/365	1.07 (0.90–1.29)	315/531	0.97 (0.75–1.19)	388/531	0.98 (0.79–1.26)
Sex												
Male	159/243	Reference	188/243	Reference	311/544	Reference	384/544	Reference	470/787	Reference	572/787	Reference
Female	49/83	1.17 ((0.93–1.46)	60/83	1.24 (0.98–1.84)	79/160	0.80 (0.62–1.04)	100/160	0.78 (0.63–1.03)	128/243	1.05 (0.77–1.38)	160/243	0.95 (0.70–1.32)
Tumor site												
Proximal	59/98	Reference	67/98	Reference	115/196	Reference	136/196	Reference	174/294	Reference	203/294	Reference
Body	72/102	1.08 ((0.85–1.23)	84/102	1.21 (0.94–1.57)	140/257	0.97 (0.77–1.29)	173/257	1.05 (0.84–1.33)	212/361	0.98 (0.82–1.29)	257/361	0.92 (0.66–1.35)
Distal	77/126	1.06 (0.88–1.37)	97/126	1.09 (0.88–1.39)	135/251	0.94 (0.72–1.28)	175/251	1.16 (0.93–1.46)	212/375	0.96 (0.85–1.24)	272/375	0.93 (0.68–1.32)
Lauren classification^b^											
Intestinal	79/147	Reference	91/147	Reference	135/293	Reference	169/293	Reference	214/440	Reference	260/440	Reference
Diffuse	123/170	1.43 (1.05–1.88)	150/170	1.55 (1.12–2.17)	244/391	1.55 (1.08–1.89)	301/391	1.59 (1.12–1.82)	367/561	1.48 (1.06–1.99)	451/561	1.56 (1.10–1.98)
Differentiation^b^												
Well/moderate	92/168	Reference	110/168	Reference	163/354	Reference	215/354	Reference	255/522	Reference	325/522	Reference
Poor	112/152	1.44 (1.02–1.82)	133/152	1.43 (1.01–1.93)	219/336	1.58 (1.21–1.83)	259/336	1.61 (1.24– 1.96)	331/488	1.61 (1.14–2.07)	392/488	1.68 (1.22–2.46)
TNM stage												
I	21/56	Reference	29/56	Reference	61/147	Reference	76/147	Reference	82/203	Reference	105/203	Reference
II	83/143	1.96 (1.20–3.21)	101/143	2.12 (1.38–3.25)	186/339	1.47 (1.08–2.01)	232/339	1.57 (1.19–2.08)	269/482	1.58 (1.14–2.38)	333/482	1.65 (1.21–2.47)
III	77/93	3.62 (2.16–6.05)	84/93	4.17 (2.63–6.62)	102/163	1.82 (1.28–2.58)	129/163	2.11 (1.54–2.88)	179/256	2.22 (1.29–3.63)	213/256	2.99 (1.57–4.79)
IV	27/34	4.20 (2.32–7.62)	34/34	11.39 (6.53–19.89)	41/55	2.27 (1.50–3.43)	47/55	2.38 (1.62–3.48)	68/89	3.58 (1.64–5.52)	81/89	5.86 (1.93–9.27)
ACT^c^												
No	44/68	Reference	62/68	Reference	68/92	Reference	73/92	Reference	112/160	Reference	135/160	Reference
Yes	116/168	0.79 (0.53–0.99)	123/168	0.53 (0.39–0.72)	220/410	0.58 (0.44–0.76)	288/410	0.68 (0.52–0.88)	336/578	0.68 (0.54–0.84)	411/578	0.63 (0.52–0.76)

Note: HR indicates hazard ratio; CI, confidence interval; ACT, adjuvant chemotherapy.

^a^Adjusted by age, sex, tumor site, Lauren classification, differentiation, TNM stage, and ACT where appropriate.

^b^Other classification and unknown differentiation were censored due to the small number of subjects in this subgroup.

^c^Only including stage II and stage III GC patients received FOLFOX-based ACT.

### Prognostic analysis of *SSBP1* SNPs in GC patients

**Figure 1:**
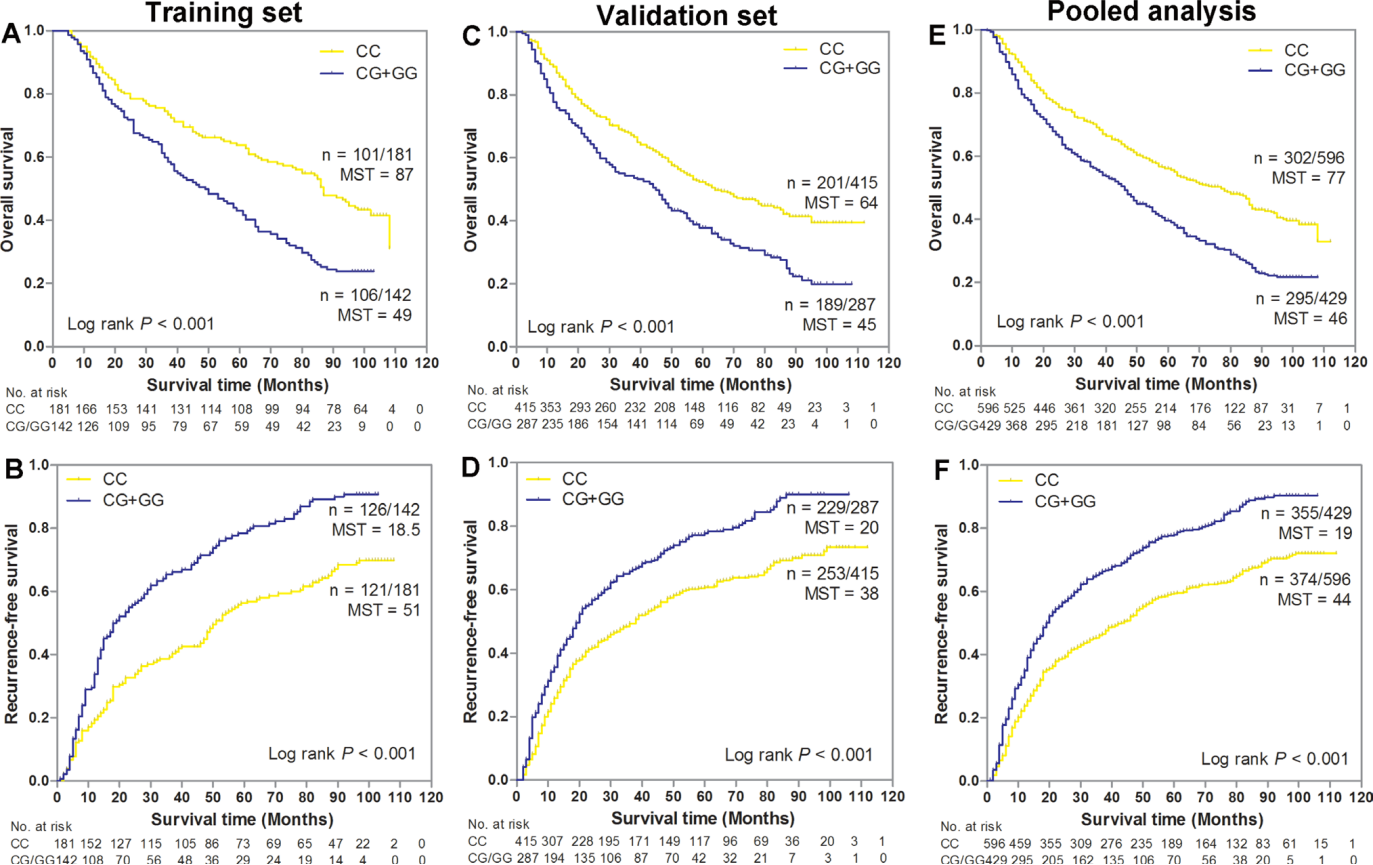
Kaplan-Meier estimates of overall survival (OS) and recurrence-free survival (RFS) for gastric cancer (GC) patients stratified by SNP rs6976500 genotypes in (**A**–**B**) training set, (**C**–**D**) validation set and (**E**–**F**) pooled analysis. MST indicates median event-free survival times (in months). Patient numbers may not add up to 100% of available subjects because of missing genotyping data.

We evaluated the associations of potential functional SNPs in *SSBP1* with GC survival in three genetic models using Cox regression analyses (Table 2). Our data showed that SNP rs6976500 exhibited statistically significant associations with OS and RFS of GC patients under dominant model in training cohort. Patients carrying variant-containing (CG/GG) genotypes had a significant increase risk of OS (HR = 1.42, 95% CI = 1.15–1.75, *P* < 0.001) and RFS (HR = 1.38, 95% CI = 1.03–.86, *P* = 0.031) when compared with those carrying homozygous wild-type (CC) genotype. Kaplan-Meier curves analysis further provided a significantly difference in the risk of OS between the homozygous wild (CC) genotypes and the variant-containing (CG/GG) genotypes (*P* < 0.001, Figure 1A). The median OS time was 87 months in patients with the CC genotype and 49 months in patients with CG/GG genotypes. Similarly, patients carrying heterozygous variant (CG/GG) genotypes of rs6976500 had a poorer RFS than did those carrying homozygous wild (CC) genotype (*P* < 0.001, Figure 1B). The median RFS time was 18.5 months in patients with the CG/GG genotypes and 51 months in patients with CC genotype. These results indicated a detrimental effects of variant-containing (CG/GG) genotypes of rs6976500 on GC death and recurrence risk.

**Table 2:** Association of SSBP1 SNPs and clinical outcome of gastric cancer patients

SNP location nucleotide change	Genotype		Training set			Validation set			Pooled analysis		
			Events^a^/Total	HR^b^ (95% CI)	*P*	Events^a^/Total	HR^b^ (95% CI)	*P*	Events^a^/Total	HR^b^ (95% CI)	*P*
Overall survival										
rs6976500		CC	101/181	Reference		201/415	Reference		302/596	Reference	
promoter		CG	89/120	1.40 (1.04–1.90)	**0.028**	161/252	1.38 (1.11–1.71)	**0.004**	250/372	1.39 (1.17–1.65)	**0.001**
–912 C>G		GG	17/22	1.26 (0.74–2.16)	0.398	28/35	1.76 (1.17–2.65)	**0.008**	45/57	1.56 (1.13–2.15)	**0.007**
		Additive		1.22 (0.98–1.52)	**0.077**		1.35 (1.14–1.59)	**0.001**		1.31 (1.15–1.49)	**0.001**
		Dominant		1.38 (1.03–1.86)	**0.031**		1.42 (1.15- 1.75)	**<0.001**		1.41 (1.19–1.67)	**<0.001**
		Recessive		1.05 (0.63–1.75)	0.855		1.51 (1.02–2.23)	**0.039**		1.32 (0.97–1.80)	0.076
rs12670074		TT	154/241	Reference		283/516	Reference		437/757	Reference	
promoter		TC	48/76	0.92 (0.66–1.28)	0.615	96/168	1.05 (0.84–1.33)	0.654	144/244	1.01 (0.83–1.22)	0.935
228 T>C		CC	4/6	1.19 (0.43–3.30)	0.737	9/17	0.74 (0.38–1.44)	0.379	13/23	0.84 (0.48–1.46)	0.533
		Additive		0.96 (0.72- 1.28)	0.785		0.98 (0.81–1.19)	0.860		0.98 (0.83–1.15)	0.781
		Dominant		0.94 (0.68–1.29)	0.685		1.02 (0.81–1.28)	0.873		0.99 (0.83–1.19)	0.927
		Recessive		1.21 (0.44–3.34)	0.713		0.73 (0.38–1.42)	0.354		0.84 (0.48–1.45)	0.527
Recurrence-free survival										
rs6976500		CC	121/181	Reference		253/415	Reference		374/596	Reference	
promoter		CG	105/120	1.62 (1.23–2.14)	**0.001**	199/252	1.44 (1.19–1.75)	**0.001**	304/372	1.49 (1.17–1.78)	**0.001**
–912 C>G		GG	21/22	1.73 (1.06–2.83)	**0.028**	30/35	1.56 (1.05–2.31)	**0.025**	51/57	1.64 (1.13–2.22)	**0.002**
		Additive		1.42 (1.16–1.74)	**0.001**		1.34 (1.15–1.56)	**0.001**		1.37 (1.14–1.54)	**0.001**
		Dominant		1.63 (1.25–2.14)	**<0.001**		1.46 (1.21–1.76)	**<0.001**		1.50 (1.29–1.75)	**<0.001**
		Recessive		1.34 (0.84–2.13)	0.218		1.30 (0.89–1.90)	0.168		1.35 (1.01–1.80)	**0.045**
rs12670074		TT	183/241	Reference		354/516	Reference		538/757	Reference	
promoter		TC	57/76	0.91 (0.67–1.23)	0.537	113/168	1.02 (0.82–1.26)	0.858	170/244	0.97 (0.82–1.16)	0.767
228 T>C		CC	4/6	1.04 (0.38–2.87)	0.945	14/17	1.07 (0.63–1.83)	0.810	18/23	1.05 (0.66–1.69)	0.837
		Additive		0.93 (0.71–1.22)	0.616		1.03 (0.86–1.22)	0.784		0.99 (0.86–1.15)	0.898
		Dominant		0.97 (0.68–1.23)	0.563		1.03 (0.84–1.26)	0.815		0.98 (0.83–1.16)	0.821
		Recessive		1.05 (0.38–2.91)	0.920		1.06 (0.63–1.81)	0.823		1.06 (0.66–1.69)	0.815

Note: HR indicates hazard ratio; CI, confidence interval. Significant *P* values were presented in bold.

^a^Numbers may not add up to 100% of available subjects because of missing genotyping data.

^b^Adjusted by age, sex, tumor site, Lauren classification, differentiation, TNM stage, and ACT where appropriate.

We further validated the prognostic effects of *SSBP1* polymorphisms on OS and RFS in an independent validation cohort of 704 GC patients. As expected, the risk of death and recurrence of patients with the SNP rs6976500 CG/GG genotypes in the independent validation cohort were significantly elevated than those with the SNP rs6976500 CC genotype (HR = 1.42, 95% CI = 1.15–1.75; HR = 1.46, 95% CI = 1.21–1.76, respectively) (Table 2). Combining two patient cohorts, pooled analysis also confirmed the previously observed associations with OS (HR = 1.41, 95% CI = 1.19–1.67) and RFS (HR = 1.50, 95% CI = 1.29–1.75) in GC patients. Kaplan-Meier survival curves analysis showed that patients carrying rs6976500 CG/GG genotypes had both significantly shorter OS and RFS than those carrying rs6976500 CC genotype in the independent validation cohort (both *P* < 0.001, Figure 1C and 1D) and pooled analysis (both *P* < 0.001, Figure 1E and 1F). The median OS time and RFS time of patients with the rs6976500 CG/GG genotypes were significantly shorter than those with the CC genotype in the independent validation cohort (45 months *vs.* 64 months; 20 months *vs.* 38 months, respectively) and pooled analysis (46 months *vs.* 77 months; 19 months *vs.* 44 months, respectively). All above evidences suggested that the rs6976500 genotypes were independent prognostic indicators for GC patients. In addition, we did not observe any association of SNP rs12670074 with OS and RFS in GC patients (Table 2 and Supplementary Figure 2).

### Stratified analysis of prognostic significance of SNP rs6976500 genotypes by host characteristics

**Figure 2:**
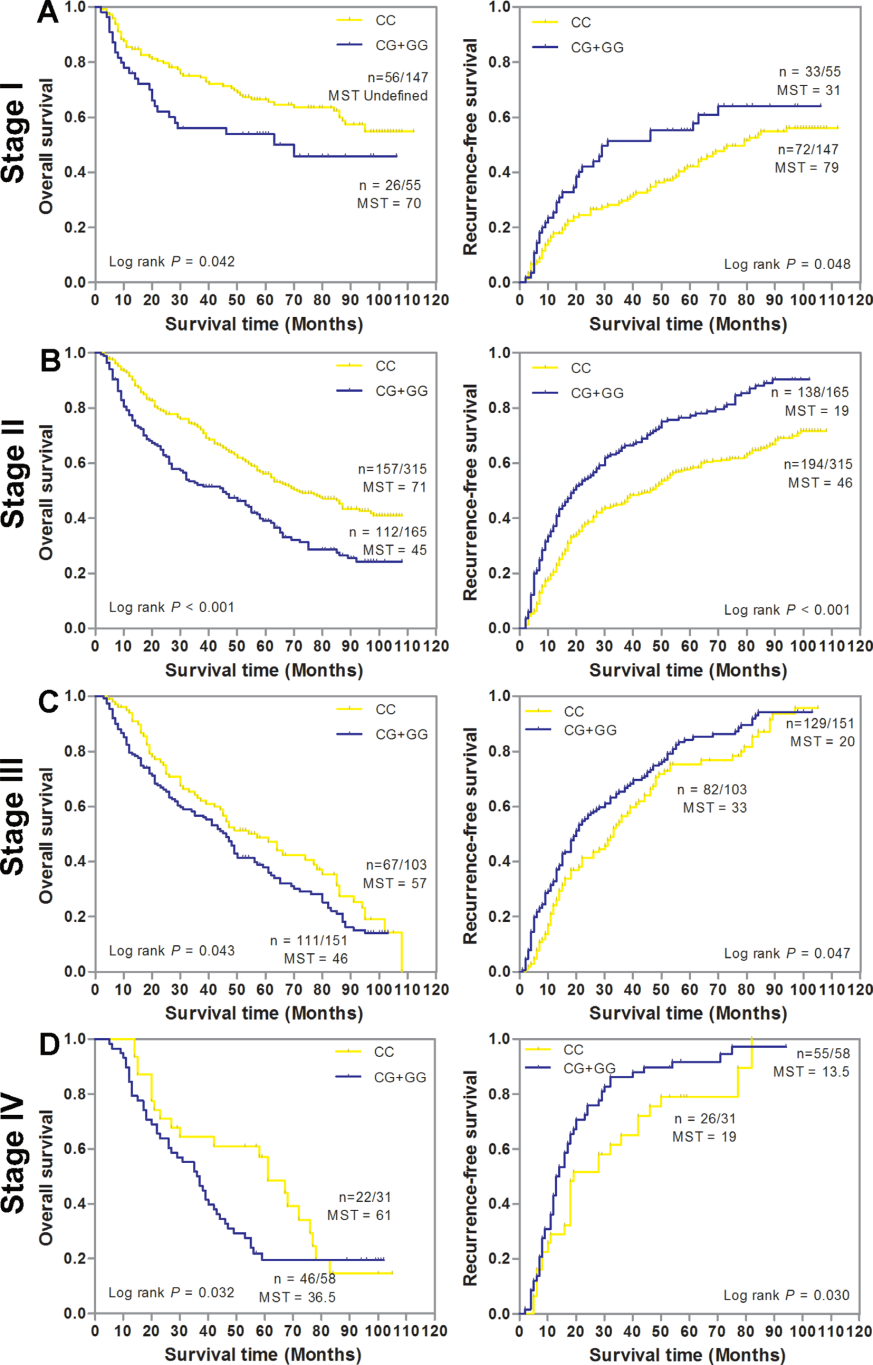
Kaplan-Meier estimates of overall survival (OS) and recurrence-free survival (RFS) according to SNP rs6976500 genotypes differential stages of GC patients in the combination of training and validation sets (**A**) Stage I (*n* = 202), (**B**) Stage II (*n* = 580), (**C**) Stage III (*n* = 254), (**D**) Stage IV (*n* = 89). MST indicates median event-free survival times (in months). Patient numbers may not add up to 100% of available subjects because of missing genotyping data.

**Figure 3:**
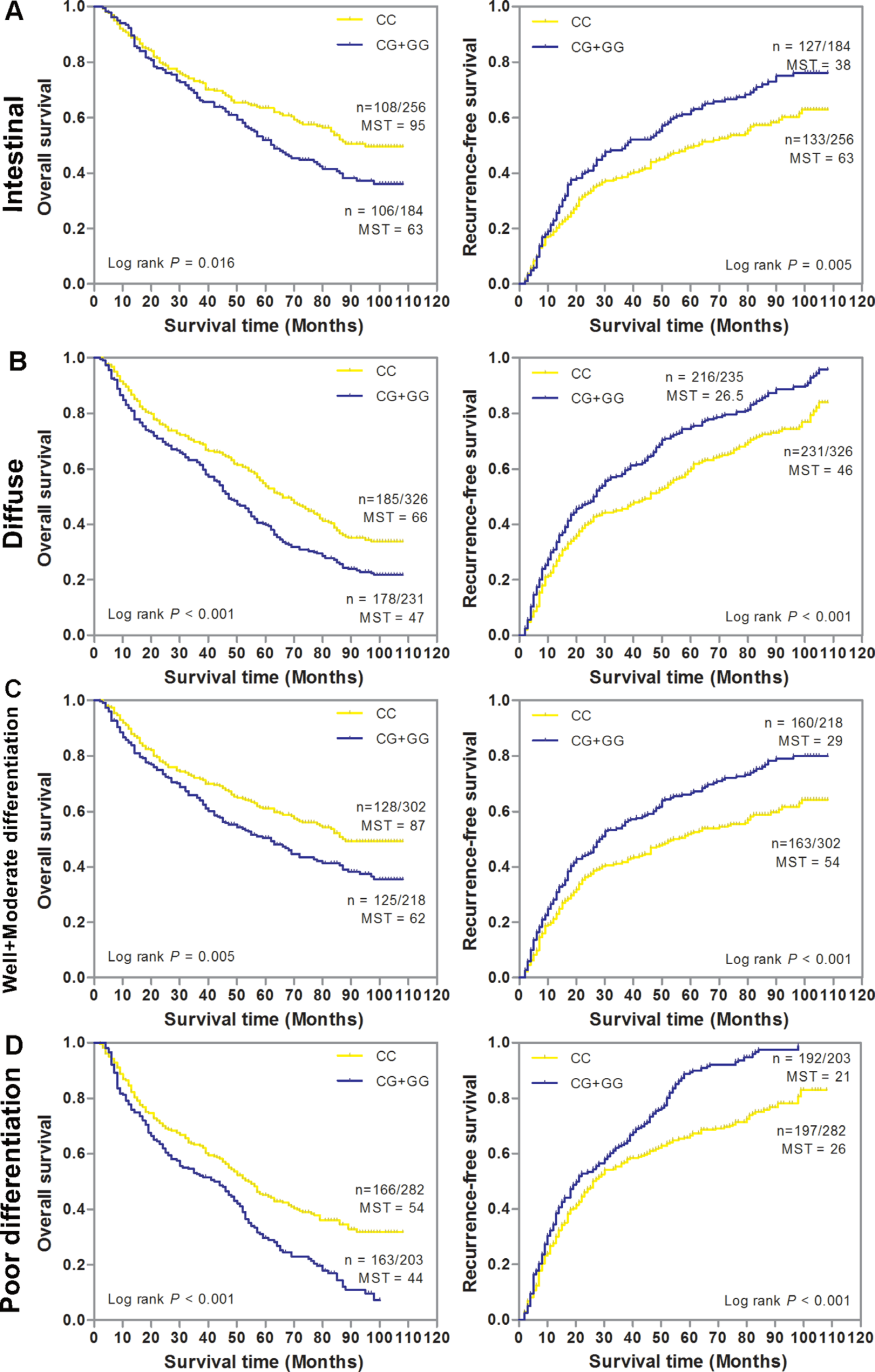
Kaplan-Meier estimates of overall survival (OS) and recurrence-free survival (RFS) according to SNP rs6976500 genotypes differential histological types or Lauren classification of GC patients in the combination of training and validation sets (**A**) Intestinal (*n* = 460), (**B**) Diffuse (*n* = 557), (**C**) Well and moderate differentiation (*n* = 520), (**D**) Poor differentiation (*n* = 485). MST indicates median event-free survival times (in months). Patient numbers may not add up to 100% of available subjects because of missing genotyping data.

We first conducted a stage-stratified analysis to evaluate whether SNP rs6976500 can predict GC patients’ prognosis within each clinical TNM stage stratum (stage I, II, III and IV) in the combined GC patients. Kaplan-Meier survival analysis showed that the significant detrimental effects of variant-containing (CG/GG) genotypes on OS and RFS were almost observed in each stage stratum of GC patients. For details, patients with CG/GG genotypes had significantly worse OS and RFS than those with homozygous wild (CC) genotype (*P* < 0.05, for all) in each stage stratum (Figure 2A–2D). Meanwhile, we also assessed the prognostic value of SNP rs6976500 in the combined GC patient stratified by histological differentiation and Lauren classification. As shown in Figure 3A–3D, the significant increased risk of OS and RFS conferred by SNP rs6976500 CG/GG genotypes were observed in each differentiation subgroup (well plus moderate differentiated or poor differentiated) or Lauren classification subgroup (intestinal or diffuse) (*P* < 0.05, for all), suggesting that SNP rs6976500 genotypes can predict prognosis of GC patients within each differentiation subtype and Lauren classification. In addition, we also evaluated the associations between rs6976500 genotypes and GC survival in stratified analysis by age, sex and tumor site, and found that the associations was significant in each subgroup (Supplementary Table 2).

### SNP rs6976500 genotypes complementing to TNM staging prognostication

**Figure 4:**
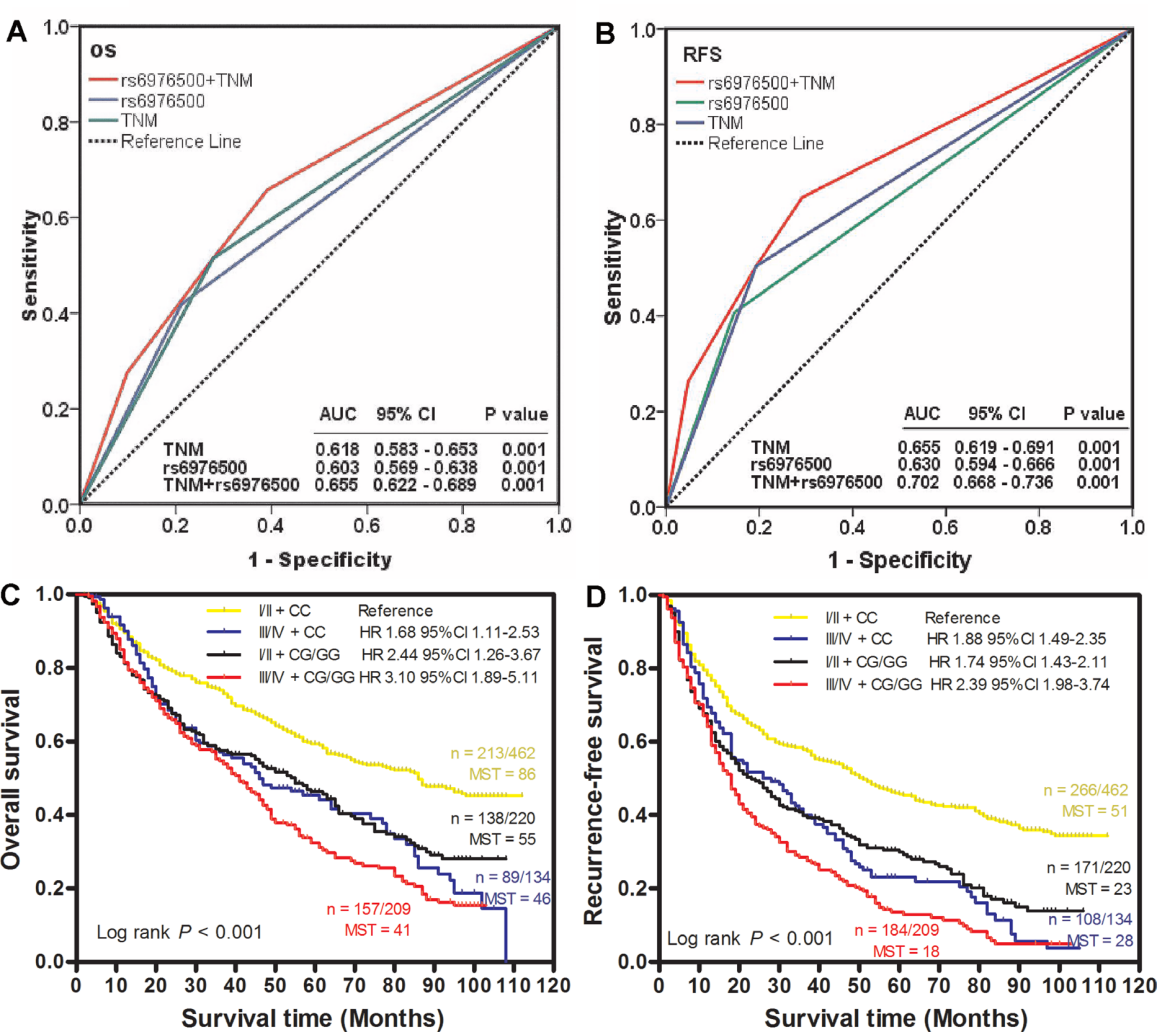
Joint prognostic value of SNP rs6976500 genotypes and TNM stage in GC patients ROC analysis showed that combined rs6976500 genotypes and TNM stage model had a better prediction value than did rs6976500 genotypes alone or TNM stage alone model in both OS (**A**) and RFS (**B**). (**C** and **D**) Kaplan-Meier curves of OS and RFS for GC patients subgrouped by rs6976500 genotypes and TNM stage. Hazards ratios and 95% CIs were calculated by multivariate Cox proportional hazards regression model, adjusted for age, sex, tumor site, differentiation, Lauren classification, TNM stage, adjuvant chemotherapy and rs6976500 genotypes as covariates. MST indicates median event-free survival times (in months). Patient numbers may not add up to 100% of available subjects because of missing genotyping data.

To assess whether rs6976500 genotypes could provide additional predictive abilities for clinical TNM stage in combined GC patients, the ROC curve analysis was performed based on TNM stage alone, rs6976500 genotypes alone, and TNM stage plus rs6976500 genotypes. Through comparing the area under curve (AUC), the combination of clinical TNM staging system and rs6976500 genotypes had the largest AUC, indicating a significantly better predictive ability for OS and RFS than TNM stage alone and rs6976500 genotypes alone (Figure 4A and 4B). Then, Kaplan-Meier curves were used to compare different RFS and OS between different GC patient subgroups stratified by clinical TNM stage plus rs6976500 genotypes. Our data showed that patients carrying rs6976500 CG/GG genotypes in stage III and IV had the worst RFS and OS, whereas those carrying rs6976500 CC genotype in stage I and II exhibited the best RFS and OS (both log-rank *P* < 0.001) (Figure 4C and 4D).

For details, the AUC and 95% CIs of TNM stage for OS and RFS were 0.618 (0.583–0.653) and 0.655 (0.619–0.691), respectively, and that of the rs6976500 genotypes were 0.603 (0.569–0.638) and 0.630 (0.594–0.666); whereas that of TNM stage plus rs6976500 genotypes were 0.655 (0.622–0.689) and 0.702 (0.668–0.736), a significant improvement over TNM stage alone or rs6976500 genotypes alone (Figure 4A and 4B). Furthermore, multivariate Cox analysis was performed and found that patients carrying rs6976500 CG/GG genotypes at stage III and IV exhibited the highest risk of death (HR = 4.94, 95% CI = 1.96– 8.73; *P* < 0.001) and recurrence (HR = 4.49, 95% CI = 1.58–8.17; *P* < 0.001) when using patients carrying rs6976500 CC genotype at stage I and II as reference (Figure 4C and 4D). Collectively, these data indicated that integration of rs6976500 genotypes and clinical TNM staging system could improve the predictive abilities for OS and RFS in predicting GC prognosis.

### Modulating effect of SNP rs6976500 on the prognostic significance of ACT

FOLFOX-based ACT was recommended as first-line therapy for patients with stage II and III GC after surgery in the NCCN Guidelines^®^ [19]. However, in clinical, which patients will absolute benefit and the magnitude of benefit of ACT remain unclear. In present study, all stage IV patients received FOLFOX-based ACT after surgery, whereas, in stage I, none of these patients underwent postsurgical ACT. In order to eliminate confounding effect of tumor stages, only stage II and III GC patients were selected to evaluate the predictive effects of the rs6976500 genotypes for OS and RFS benefit from FOLFOX-based ACT. In addition, we excluded 18 patients who received non-FOLFOX regimen. Finally, we had 578 stage II and III patients received FOLFOX-based ACT for the downstream analyses. We first performed a stratified analysis by ACT to evaluate whether the FOLFOX-based ACT modulates the prognostic significance of rs6976500. Our data showed that patients carrying rs6976500 CG/GG genotypes still had significantly elevated risk of death and recurrence in each subgroup except for OS in validation cohort (Supplementary Table 3). Whereas, these significant relationships between rs6976500 CG/GG genotypes and GC prognosis were more prominent in patients without FOLFOX-based ACT in comparison to those with FOLFOX-based ACT, suggesting a potential modulating effect between ACT and SNP rs6976500 genotypes on GC survival.

In order to further assess whether the association between ACT and GC outcomes was also modulated by SNP rs6976500, we conducted stratified and interaction analyses of ACT with rs6976500. As shown in Table 3, significant interactions were identified in training cohort (*P*_interaction_ = 0.024 and < 0.001 for OS and RFS), validation cohort (*P*_interaction_ = 0.003 and 0.026 for OS and RFS), as well as in pooled analysis (*P*_interaction_ = 0.015 and 0.002 for OS and RFS). The risk of recurrence and death of patients received FOLFOX-based ACT were significantly reduced than those without FOLFOX-based ACT in training cohort (HR: 0.79 *vs*. 0.53,), validation cohort (HR: 0.58 *vs.* 0.68) and in pooled analysis (HR: 0.68 *vs*. 0.63). However, these significant protective effects of ACT only exhibited in patients carrying rs6976500 CG/GG genotypes, but not in those carrying CC genotype (Table 3). This subgroup analysis revealed that only patients carrying rs6976500 CG/GG genotypes could benefit from FOLFOX-based ACT, indicating SNP rs6976500 may be a promising prognostic indicator of response to FOLFOX-based ACT for GC patients.

**Table 3:** Modulating effects of adjuvant chemotherapy (ACT) on gastric cancer (GC) clinical outcome stratified by SNP rs6976500 in SSBP1 gene

rs6976500 genotype^b^	ACT	Training set			Validation set			Pooled analysis		
		Events/Total^a^	HR (95% CI)^c^	*P*	Events/Total^a^	HR (95% CI)^c^	*P*	Events/Total^a^	HR (95% CI)^c^	*P*
Overall Survival										
In all patients	No ACT	44/68	Reference		68/92	Reference		112/160	Reference	
	ACT	116/168	0.79 (0.53–0.99)	**0.047**	220/410	0.58 (0.44–0.76)	**0.001**	336/578	0.68 (0.54–0.84)	**0.001**
CC	No ACT	27/45	Reference		25/39	Reference		52/84	Reference	
	ACT	53/85	0.75 (0.47–1.20)	0.232	129/249	0.86 (0.65–1.31)	0.246	182/334	0.88 (0.65–1.20)	0.428
CG/GG	No ACT	16/22	Reference		43/53	Reference		59/75	Reference	
	ACT	63/82	0.65 (0.42–0.88)	**0.016**	91/159	0.39 (0.26–0.57)	**<0.001**	154/241	0.51 (0.38–0.69)	**<0.001**
			*P*_interaction_	**0.024**		*P*_interaction_	**0.003**		*P*_interaction_	**0.015**
Recurrence-free Survival										
In all patients	No ACT	62/68	Reference		73/92	Reference		135/160	Reference	
	ACT	123/168	0.53 (0.39–0.72)	**<0.001**	288/410	0.68 (0.52–0.88)	**0.003**	411/578	0.63 (0.52–0.76)	**0.001**
CC	No ACT	39/45	Reference		30/39	Reference		69/84	Reference	
	ACT	54/85	0.62 (0.35–1.01)	0.058	163/249	0.83 (0.54–1.39)	0.221	217/334	0.75 (0.57–1.01)	0.063
CG/GG	No ACT	22/22	Reference		43/53	Reference		65/75	Reference	
	ACT	69/82	0.49 (0.30–0.79)	**0.004**	123/159	0.38 (0.23–0.56)	**<0.001**	192/241	0.44 (0.33–0.59)	**<0.001**
			*P*_interaction_	**<0.001**		*P*_interaction_	**0.026**		*P*_interaction_	**0.002**

Note: HR, hazard ratio; CI, confidence interval. Significant *P* values were presented in bold.

^a^Only including stage II and stage III GC patients received FOLFOX-based ACT.

^b^Numbers may not add up to 100% of available subjects because of missing genotyping data.

^c^Adjusted by age, sex, tumor site, tumor size, differentiation, and TNM stage where appropriate.

### Effects of rs6976500 genotypes on the promoter activity and expression levels of *SSBP1* in GCs

SNP rs6976500 is located at -912 position in the 5′-UTR of *SSBP1* gene. Previous evidence has suggested that SNP in the 5′ flanking region can alter gene expression by impacting on TF binding regulations [20]. Based on ALGGEN PROMO software analysis (http://alggen.lsi.upc.edu/recerca/menu_recerca.html), 13 potential TFBSs have been predicted very close to the actual rs6976500 SNP position (Supplementary Table 4). Therefore, SNP rs6976500 might contribute to promoter transcriptional activity and gene expression of *SSBP1* by affecting TF regulations.

**Figure 5:**
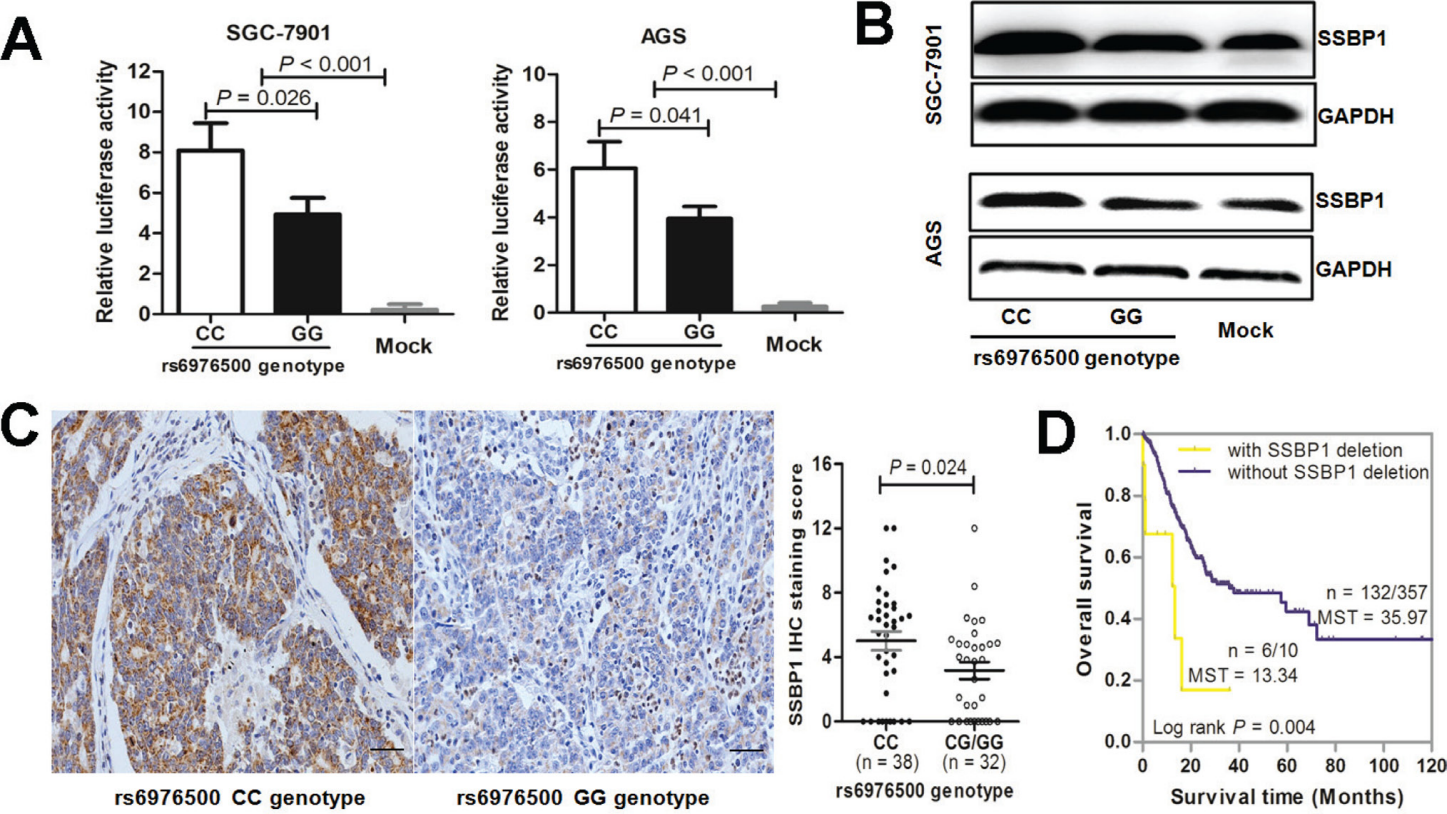
Effects of SNP rs6976500 genotypes on the transcriptional activities and expression levels of SSBP1 in GCs (**A**) Comparison of luciferase activities in SGC-7901 and AGS cells transfected with promoter reporter constructs containing rs6976500 C allele (pGL3-SSBP1-C) or G allele (pGL3-SSBP1-C). The transcriptional activity in SGC-7901 and AGS cells transfected with pGL3-SSBP1-C was much high than that of pGL3-SSBP1-G and pGL3-Basic plasmid (mock control). (**B**) Western blot demonstrated that the SSBP1 protein level of GC cells transfected with pGL3-SSBP1-C was significantly increased compared with those transfected with pGL3-SSBP1-G or mock control. (**C**) Representative picture of immunohistochemical staining, much stronger positive staining for SSBP1 was detected in GCs with rs6976500 wild-type genotype (CC) compared with homozygous variant genotype (GG); magnification ×200. (**D**) Kaplan-Meier estimates of OS for GC patients stratified by SSBP1 gene deletion or not in the TCGA cohort (*P* = 0.004; log-rank test).

To test this hypothesis, two stable GC cell lines were established by transfecting with reconstructed plasmids pGL3-SSBP1-C or pGL3-SSBP1-G. Empty pGL3-Basic vector transfectants served as negative control. Then, a dual-luciferase reporter assay was performed. As shown in Figure 5A, we found that both reconstructed reporter plasmids pGL3-SSBP1-C and pGL3-SSBP1-G drove significantly higher luciferase expression than that of the negative control (all *P* < 0.001 in SGC7901 and AGS GC cell line). Moreover, the luciferase expression of the pGL3-SSBP1-G plasmid was significantly reduced than that of pGL3-SSBP1-C plasmid in both SGC-7901 cells (*P* = 0.026) and AGS cells (*P* = 0.041). This suggests that the SNP rs6976500 G allele could significantly attenuate the promoter activity of *SSBP1* gene.

Then, western blot assay was used to evaluate the protein level of SSBP1 in two stable GC cell lines carrying different rs6976500 genotypes for further confirming our previous findings. The SSBP1 protein level in GC cells (SGC-7901 and AGS) transfected with the pGL3-SSBP1-C plasmid was significantly increased in comparison to the one with pGL3-SSBP1-G plasmid or mock control. However, the SSBP1 protein level had no significant difference between GC cells transfected with pGL3-SSBP1-G and mock control (Figure 5B). Moreover, we investigated the SNP rs6976500 genotypes and SSBP1 protein expression in another cohort of 70 primary GCs by immunohistochemistry (IHC). The characteristics of GC patient in this cohort was listed in Supplementary Table 5. Our results revealed that the percentage of positive SSBP1 staining of tumor tissues had no significant difference between GC patients with homozygous wild (CC) genotype and those carrying variant-containing (CG and GG) genotypes (9/38 *vs.* 10/32, *P* = 0.403), but the SSBP1 expression level in GCs with homozygous wild (CC) genotype was significantly elevated in comparison to those with the variant-containing (CG/GG) genotypes (4.68 ± 0.58 *vs.* 3.03 ± 0.53, *P* = 0.042) (Figure 5C). Collectively, these data suggested that the rs6976500 G allele could significantly reduce SSBP1 gene and protein expressions. In addition, on the basis of data from the TCGA cohort, GC patients with *SSBP1* gene deletion had significantly shorter survival than those without *SSBP1* gene deletion (Figure 5D), which indirectly indicating that decreased expression of *SSBP1* gene might contribute to poor prognosis in GC.

## DISCUSSION

SSBP1 has long been regarded as a critical DNA repair protein, however, it is likely to be involved in numerous cellular biological processes, including energy metabolism [21, 22], checkpoint activation [23], genomic instability [24, 25], and radio- and chemo-sensitivity [13, 18]. Given the functional diversity of SSBP1, it has been speculated to be participated in tumor initiation and progression. Actually, in current study, we for the first time presented the compelling evidence that *SSBP1* genetic alterations significantly correlated with poor prognosis of GC. Consistently, a number of scientific studies have suggested that aberrant *SSBP1* expression is correlated to the aggressive phenotype and poor survival of human cancers. A recent report has suggested that SSBP1 acts as a tumor suppressor to restrain mammary epithelial cell phenotype transdifferentiation and breast cancer metastasis, and thereby its decreased expression contribute to the worse survival of breast cancer patients [15]. Similarly, a markedly decline in *SSBP1* expression was observed in lung cancer and down-regulation of *SSBP1* significantly increased radio-sensitivity in cancer cells [18]. In contrast, *SSBP1* expression is dramatically increased in hepatocellular carcinoma (HCC) and associated with poor prognosis [14]. Consistent with HCC, Wu *et al.* have reported that the expression of *SSBP1* is highly abundant in colorectal cancer (CRC) and closely related with poor outcomes of CRC patients [26]. Apart from that, a study showed that SSBP1 expression was also elevated in human osteosarcoma cells and the expression level correlated with an aggressive phenotype [16]. These inconsistent results indicated that the role of SSBP1 in tumor progression may be cancer organ-specific. The underlying mechanisms has been partially investigated. Several previous studies have demonstrated that SSBP1 decrease or deletion may lead to chromosome instability [11], and eventually prompt tumorigenesis [27], conversely, overexpression of SSBP1 also may contribute to tumor initiation through increasing mtDNA replication, thus leading to greater energy supplying and enhanced immunosuppression to facilitate tumor growth [28]. Collectively, these researches highlighted the vital role of SSBP1 in tumor initiation and progression, making SSBP1 an attractive tumor biomarker.

SNPs, at a specific base position in the human genome, can alter gene expression and protein functions [29]. Despite the widely investigations of SSBP1 expression on cancer evolution, however, there was no study investigating the effect of *SSBP1* gene polymorphisms on GC prognosis or ACT treatment response. In this study, we observed that the SNP rs6976500 within *SSBP1* gene was closely related to increased OS and RFS in GC patients. This finding from the training cohort was confirmed by the results obtained from the independent validation cohort, which indicated a significantly better OS and RFS in patients carrying CC genotype than those carrying CG/GG genotypes. This finding is biologically plausible because SNP rs6976500 lies in the 5′ untranslated region of *SSBP1* gene and hence have a potential influence on gene expression. However, the molecular functions of the SNP rs6976500 has never been investigated. In present study, we first performed a bioinformatics analysis using ALGGEN PROMO software, 13 potential TFBSs have been predicted very close to the actual rs6976500 SNP position, indicating that SNP rs6976500 may influence promoter transcriptional activity and gene expression of *SSBP1* by affecting TF regulations [20]. Our functional experiments have demonstrated that the G allele of SNP rs6976500 could significantly attenuate *SSBP1* promoter activity and reduce expression of *SSBP1* in GC cells. We further assessed the expression of *SSBP1* in 70 GC tissues with SNP rs6976500 genotype data and found that the expression of *SSBP1* in tissues carrying CG/GG genotypes was significantly lower than those with CC genotype, suggesting that the detrimental effect of rs6976500 CG/GG genotypes was correlated to down-regulation of *SSBP1* expression. Although there is no direct evidence showing decreased *SSBP1* expression in cancerous tissues contribute to worse GC survival, however, from TCGA cohort, GC patients with *SSBP1* gene deletion had significantly shorter survival than those without *SSBP1* deletion, suggesting the potential importance of lower SSBP1 expression in progression of GC. And more notably, the lower expression of SSBP1 in gastric cancerous tissues indicated the biological plausibility, for the G allele of rs6976500 was associated with the worse prognosis of GC, and drove significantly lower leuciferase expression. Collectively, these evidences strongly indicated a direct causal role of rs6976500 on GC, and therefore it may be a promising prognostic biomarker and a potential therapeutic target for GC.

Of particular concern, SNP rs6976500 of *SSBP1* was found to be associated with increased OS and RFS in GC, especially its modulating effect on the widespread use of FOLFOX-based ACT. Nowadays, FOLFOX-based ACT has been recommended as a first-line postoperative adjuvant therapy after curative intent surgical resection in advanced GC [19]. However, the absolute benefit of FOLFOX-based ACT for GC patients remains controversial. Previous studies have suggested that genomic polymorphisms can influence drug transport, metabolism and cellular response, and lead to individual variations in terms of the response and toxicity and even to overall survival [30, 31]. Up to now, a series of researches have been carried out to explore the associations between treatment response of GC patients and individual genetic polymorphisms which will determine the efficacies and toxicities of chemotherapeutic agents, especially of 5-FU and platinum agents [32]. Several studies have reported that SNPs in ERCC, XPD and XRCC genes could change the activities of platinum drugs, thereby affecting cancer patients’ survival [33–35]. In addition, polymorphism within GSTP1 gene is involved in platinum detoxification and significantly associated with better survival in cisplatinum-treated GC [36]. We thus performed a stratified analysis by ACT to evaluate whether the FOLFOX-based ACT modulates the prognostic significance of SNP rs6976500. Our data showed that the risk of death and recurrence conferred by SSBP1 rs6976500 CG/GG genotypes were more prominent in GC patients without receiving FOLFOX-based ACT than those with ACT, suggesting the presence of potential interaction between SNP rs6976500 and ACT for modulating GC prognosis. Therefore, a test for interaction was performed and revealed a significant interaction effect between ACT and rs6976500. The finding of this significant interaction is in accordance with the observation that the HRs of ACT in stratified analysis were opposite in patients with rs6976500 CC genotype and those with CG/GG genotypes. That is, FOLFOX-based ACT only conferred a favorable prognosis for GC patients carrying CG/GG genotypes of rs6976500, but not for those carrying CC genotype (Table 3). These findings indicated that SSBP1 rs6976500 G allele might enhance the sensitivity of GC patients to the FOLFOX-based ACT. Previous animal model study provided molecular mechanism evidence to indirectly support our findings that *SSBP1* gene inhibition led to an increased mouse cell apoptosis induced by etoposide [13]. Subsequent research revealed that knock-down of endogenous *SSBP1* dramatically enhanced the sensitivity of cervical cancer cells to chemotherapy [26]. It is therefore highly plausible that rs6976500 G allele genotype enhance the sensitivity of cancer cells to chemotherapy agents maybe through down-regulating *SSBP1* expression. However, given the modest samples size of our study population and the heterogeneity in clinical characteristics, the interaction analysis may not be adequately powered. Therefore, the results are not definitive at this point and future studies with homogeneous patient populations and larger sample size are needed to validate these findings. Nonetheless, on confirmation of our observation by large-scale studies in the future, genotyping for the *SSBP1* rs6976500 may ultimately assist in guiding treatment decisions for GC patients.

Currently, the TNM staging and WHO histologic classification are more widely used by medical professionals to predict the survival in patients with GC, but neither provides more precise prognosis [37]. Partially due to its heterogeneous nature, the GC patients, even though they have the same clinical features, have different clinical outcomes [38]. These phenomena state that current cancer staging systems, based on pathological and clinical features, have practical limitations for clinical application and there is an urgent need to identify novel molecular biomarkers that could provide supplemental information for facilitating the prognostic prediction and the better treatment decision of GC patients. Previous studies have demonstrated that several molecular biomarkers, such as gene expression profile, telomere length and genetic polymorphisms, could provide significantly supplemental prognostic value to the current cancer staging system [38]. In present study, we also found that integration of rs6976500 genotypes and TNM staging system significantly enhanced the predictive power for OS and RFS in predicting GC prognosis.

Our study has several distinct features. Firstly, the participants in this study were unrelated Chinese Han descent and enrolled from northwest area of China, which limited the confounding of ethic and geographical heterogeneity. Secondly, rs6976500 was not only suggested to be an independent prognostic indicator for GC in both stages and combined analysis, but the statistical power of each stage was sufficient enough to make the results more robust. Thirdly, we characterized the function of SNP rs6976500, making the association of this SNP with the worse survival of GC biological plausible. The major limitation of this study is that the dual-luciferase reporter assays could only indirectly support the association, the relationship between rs6976500 and SSBP1 gene expression remained to be clarified. Moreover, we could not rule out the possibility of chance findings in our study due to modest sample size and lack of external validation. In addition, patient population in present study was restricted to Han Chinese and we can not bypass the generalizability issue. Larger multi-ethnic and multicenter studies are warranted in the future.

In summary, our findings demonstrated that *SSBP1* rs6976500 was an independent prognostic marker and can improve the prognostic prediction of current TNM staging system in GC. Additionally, SNP rs6976500 might serve as a potential marker to guide treatment decisions of GC patients. Mechanically, SNP rs6976500 G allele could significantly attenuate *SSBP1* promoter activity and gene expression. Our study provides new insight into GC progression.

## MATERIALS AND METHODS

### Ethics statement

This study were approved by the Research Ethic Committee of the Fourth Military Medical University and the Medical College of Shihezi University. The procedures were performed according to the approved guidelines and to the 1964 Helsinki Declaration and its later amendments or comparable ethical standards. Each participant was voluntary and provided signed informed consent prior to taking part in present study.

### Study population

A total of 1078 Han Chinese patients who underwent radical operation for GC at Tangdu and Xijing Hospitals, affiliated to the Fourth Military Medical University (Xi’an, China) from January 2008 and June 2013 were enrolled in the present study. No patients had previous history of other cancers or blood transfusion within 3 months before operation or received any preoperative anticancer treatment. There were no age, sex, or disease stage restrictions for case recruitment. All GC patients were unrelated Chinese Han descent and newly diagnosed and histologically confirmed to be primary adenocarcinoma. In this prognosis study, we excluded 48 patients, including 37 patients who had incomplete clinical information or failed follow-up, 5 patients who died within 1 months after surgery, and 6 patients who had poor quality of sample DNA. Finally, 1030 patients who underwent radical resection for gastric adenocarcinoma were included in the present study for prognosis analysis. The remaining 1030 patients were divided into two sets: 1) the training set containing 326 patients from the Xijing Hospital of Digestive Disease between January 2008 and December 2010 was used to evaluate the prognostic significance of the selected SNPs for human GCs; 2) the independent validation set of 704 patients from the Department of General Surgery, Tangdu Hospital between July 2008 and December 2012 was used to further validate their prognostic values. Moreover, 70 *GC* tissue samples from the Department of Gastroenterology, First Affiliated Hospital of the Medical College of Shihezi University between August 2015 and June 2016 were used for evaluation of SSBP1 expression by immunohistochemistry.

### Demographic and clinical data

Demographic variables, including age, sex, ethnicity, residential region, and family history of cancer, were collected by face-to-face interviews at the time of initial visit. Detailed clinical data were collected through medical record review, or consulting with treating physicians, including time of diagnosis, time of surgery and/or chemotherapies, time of relapse and/or death, tumor stage, differentiation, histological type, tumor site, lymph node invasiveness, and treatment protocol. Tumor staging was determined according to the seventh-edition of Tumor-Node-Metastasis (TNM) Classification of the Union for International Cancer Control (UICC) and American Joint Committee on Cancer (AJCC) [39], and the lymph node invasive and organ metastasis information were included in different tumor stages. All patients with tumor stage 3 had lymph node invasion. All patients with tumor stage 4 had distant metastasis to other organs. Lauren’s criteria were used to classify the tumors into intestinal-type or diffuse-type gastric cancer [40]. The patients were followed up once in 6 months during the first two years and later on once in 12 months through telephone calling, outpatient review, or medical records. The latest follow-up data in this analysis was obtained in February 2017 and the median follow-up duration was 62 months (range 3–112 months). The rate of lost to follow up was 11.6%. Overall survival (OS) was defined as the time from surgery to GC-specific death. Recurrence-free survival (RFS) was defined as the time from surgery to the date of the first recurrence or distant metastasis of GC. Patients alive at the last follow-up were censored.

### DNA extraction, SNP selection and genotyping

For each GC patient, including 70 additional GC patients for IHC detection, 5 mL venous blood was collected before surgery and centrifuged within 30 minute. Genomic DNA was extracted from 5 mL venous blood by using the E.Z.N.A.^®^ blood DNA Midi Kit (Omega Bio-Tek, Norcross, GA, USA) in the laboratory, and then the genomic DNA was aliquoted and stored at −80°C for future analysis (1100 cases).

SNPs in *SSBP1* gene were selected using a set of web-based SNP selection tools (http://snpinfo.niehs.nih.gov/snpfunc.htm) according to the previous description [41]. Briefly, SNPs with minor allele frequency (MAF) <5% in Han Chinese population (CHB) were excluded. Potential functional SNPs were included in present study according to the following criteria: (1) SNPs in miRNA binding sites of 3′-UTR; (2) SNPs in the transcriptional factors binding site (TFBS) of the 5′-UTR (2000 bp upstream from the transcript start site); (3) SNPs in splice sites and exons. Finally, we selected 2 SNPs (rs6976500 and rs12670074) in *SSBP1* gene, both in the 5′-UTR. Genotyping was carried out using Sequenom iPLEX genotyping system (Sequenom Inc., San Diego, CA) according to the manufacturer’s protocol. Laboratory technicians who performed the genotyping were blinded to patient information. Strictly quality controls were implemented in each assay during genotyping with over 99.0% concordance with the main genotyping results. The average call rate for the SNP assay was 99.5%.

### Cell lines of human GC

Two human GC cell lines, SGC-7901 and AGS, were used in this study. These cell lines were obtained from the Cell Bank of the Chinese Academy of Sciences (Shanghai, China) where they were characterized by mycoplasma detection, DNA-Fingerprinting, isozyme detection and cell vitality detection, and they were immediately expanded and frozen such that they could be restarted every 3 to 4 months from a frozen vial of the same batch of cells. Cells were cultured in Dulbecco’s modified Eagle’s medium (DMEM) (Gibco BRL, Grand Island, NY, USA) supplemented with 10% (v/v) fetal calf serum (FBS) (Gibco BRL) at 37°C in a humidified incubator containing 5% CO_2_.

### Construction of *SSBP1* promoter-luciferase reporter plasmids with specific rs6976500 haplotypes

As shown in Supplementary Figure 1, the 2040 bp DNA sequence from –972 to +1068 position within 5′-UTR of human *SSBP1* gene, corresponding to the rs6976500-containing region, was amplified by polymerase chain reaction (PCR) using genomic DNA from carriers with rs6976500 CC or GG genotype as the template. The primer designed and synthesized by Sangon Biotech (Shanghai, China) were as follows: forward primer 5′-CGG GGT ACC ATG GTC TGC GGT ATT CAG TAC AGC CCC ATG CTG TCA GGT TTG CA-3′ with a Kpnl restriction site at the 5′-end and reverse primer 5′-GAC TCG AGT CCT CCA TAT CAA ATG AGT ACA GAG GTG GGT GGG TGA GCT -3′ with a XhoI restriction site. The PCR system consisted of 1 µL templates, 1 µL of each antiprimer, 1 µL primer, 10 µL 2× PCR Master Mix (Takara, Dalian, China) and 7 µL ddH_2_O. Thermal cycling was performed using an initial denaturation step of 96°C for 2 min, followed by 35 cycles of 94°C for 30 s, 58°C for 30 s and 72°C for 45 s, and finally 10 min at 68°C for extension. The PCR product of *SSBP1* was representative for the SNP rs6976500 C allele or G allele. The amplified fragments were isolated and purified following agrose eletrophoresis using a Gel Extract Kit (Omega Bil-Tek. Inc. Doraville, GA, USA), digested with Kpnl and XhoI (TaKaRa, Dalian, China), and ligated into the equivalent sites of the pGL3-basic vector (Promega, Madison, WI, USA) to generate the *SSBP1* promoter-reporter constructs (termed pGL3-SSBP1-C or pGL3-SSBP1-G, carrying either rs6976500 C allele or G allele). The resulting construct was confirmed by restriction enzyme digestion and DNA sequence analysis.

### Cell transfection and dual-luciferase reporter assay

The reconstructed plasmids pGL3-SSBP1-C or pGL3-SSBP1-G and the empty pGL3-Basic plasmid (225 ng/well) were transfected into human SGC-7901 or AGS cells together with the internal control pRL-CMV (25 ng/well). To generate stable cell lines constantly activating transcription of *SSBP1*, SGC-7901 and AGS cells were co-transfected with the pcDNA3.1 plasmid coding for an antibiotic resistance gene by Lipofectamine 2000 (Invitrogen, Carlsbad, CA, USA) according to the manufacturer’s instructions. Stable cell lines were selected with G418 (800 μg/ml, Sigma) and individual clones were isolated and maintained in medium containing G418 (400 μg/ml).

To measure the promoter-luciferase activities, stable GC cell lines transfected with pGL3-SSBP1-C or pGL3-SSBP1-G were harvested and luciferase activities were measured using the Dual-luciferase reporter assay system (Promega, Madison, WI, USA) according to the manufacturer’s protocol. The stable GC cells transfected with empty pGL3-Basic vector were used as mock control. The ratio of firefly to renilla luminescence was calculated for each transfectant and compared with that of blank pGL3-Basic transfected cultures. All transfections were performed three times in triplicates.

### Detection SSBP1 protein levels by western blot

To assess the effect of the SNP rs6976500 on gene expression, the protein expression levels of SSBP1 and glyceraldehyde-3-phosphate dehydrogenase (GAPDH) were evaluated by western blot. Conditioned media and cell lysate were prepared as described previously [15]. Briefly, total protein was extract from SGC-7901 cells or AGS cells using lysis buffer (containing 50mM Tris-Cl, pH 8.0, 150 mM NaCl, 0.1% SDS, 1% NP-40, 100 μg/ml PMSF) after transfection with SSBP1 promoter-luciferase reporter plasmids (either pGL3-SSBP1-C or pGL3-SSBP1-G) or empty pGL3-Basic vector. The protein concentration of the samples was measured using a bicinchoninic acid assay (BCA, Thermo Scientific) according to standard procedure. Twenty to fifty micrograms of total protein was separated via sodium dodecyl sulfate-polyacrylamide gel electrophoresis and transferred onto polyvinylidene fluoride membranes. The primary anti-SSBP1 (1:500 abcam^®^, Catalog Number ab74710, Cambridge, MA, USA) and anti-GAPDH (1:5000 Cell Signal Tech, Catalog Number 2118, Danvers, MA, USA) antibodies were used. Immunoreactive bands were captured by a ProteinSimple western blot imaging system (ProteinSimple, Santa Clara, CA, USA).

### Immunohistochemical staining for SSBP1

Formalin-fixed and paraffin-embedded tissues were sectioned for immunohistochemical staining. Following deparaffinization, 4 μm tissue sections were immersed in boiled citrate-disodium hydrogen phosphate buffer (pH = 6.0) with high pressure for 10 minutes for antigen retrieval. Sections were stained using a two-step immunoperoxidase technique performed as described [42]. A polyclonal antibody against SSBP1 (1:100 abcam^®^, Catalog Number ab74710, Cambridge, MA, USA) was used as primary antibody. Stained tissue sections were evaluated by Renli Li and Yulong Zhai, who are blinded to the clinical characteristics of the samples. Briefly, the intensity of the staining was scored using the following scale: 0, no staining of tumor cells; 1, mild staining; 2, moderate staining; 3, marked staining. The area of staining was evaluated and recorded as a percentage: 0, less than 5%; 1, 5%–25%; 2, 26%–50%; 3, 51%–75%; 4, more than 75%. The semiquantitative H score is obtained by multiplying the grades of extent and intensity of staining, ranging from 1 to 12, and graded as follows: <1, negative (−); ≥1 to <4, weak positive (+); ≥4 to <8, moderate positive (++); ≥8, strong positive (+++).

### Statistical analysis

Statistics analyses were performed using the IBM SPSS Statistics 19.0 software (IBM). Normally distributed continuous variables were expressed as the mean ± standard deviation, while abnormally distributed continuous variables were expressed as median and range. Pearson’s χ^2^-test was used to test the differences of categorical variables. Student’s *t*-test was used to analyze the difference of normally distributed continuous variables between two groups, while Mann-Whitney *U*-test was employed for the comparison of abnormally distributed continuous variables. For each SNP, three genetic model (dominant, additive and recessive models) were used for analysis. OS and RFS were compared with the Kaplan-Meier method and significance was determined by the log-rank test. The Cox proportional hazard regression model was applied to assess the effects of *SSBP1* genotypes and patients’ characteristics on OS or RFS. A receiver operating characteristic (ROC) curve was used to determine the prediction value of a parameter. Statistics significance was set at a level of 0.05 and all *P* values reported in this study were two sided.

## SUPPLEMENTARY MATERIALS FIGURES AND TABLES


