# Supplementary material for: A functional polymorphism of SSBP1 gene predicts prognosis and response to chemotherapy in resected gastric cancer patients
Source: Oncotarget. 2017 Dec 2;8(67):110861–76. doi: 10.18632/oncotarget.22864 (PMC5762290; doi:10.18632/oncotarget.22864)
Supplement: Supplementary file 1 [file oncotarget-08-110861-s001.pdf]

A functional polymorphism of *SSBP1* gene predicts prognosis and response to chemotherapy in resected gastric cancer patients

SUPPLEMENTARY MATERIALS

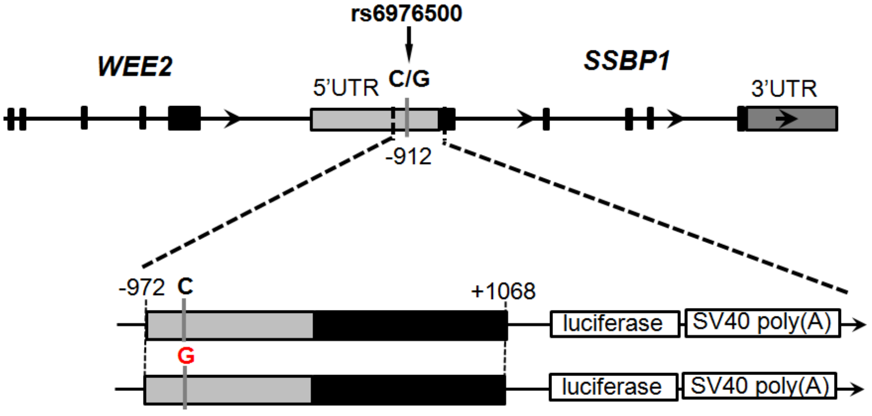

Supplementary Figure 1: Schematic representation of the human SSBP1 gene. Schematic representation is shown according to Genome Reference Consortium Human Build 38 patch release 7 (GRCh38.p7) Assembly. Arrows indicate direction of transcription. SNP rs6976500 is indicated by a vertical arrow in the 5′-UTR of SSBP1. Black boxes on the arrow line represent exons. The two types of promoter reporter constructs are shown below the *SSBP1* gene, each with the major (black) and minor/risk (red) allele listed.

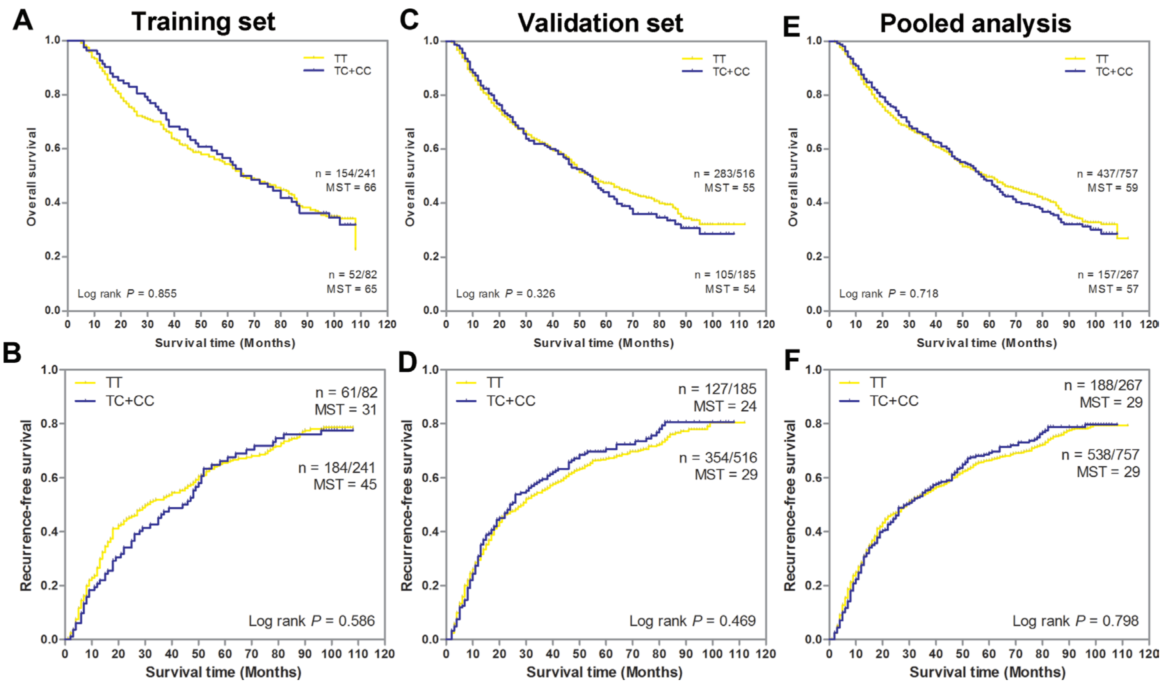

Supplementary Figure 2: Kaplan-Meier estimates of overall survival (OS) and recurrence-free survival (RFS) for gastric cancer (GC) patients stratified by SNP rs12670074 genotypes in (**A**–**B**) training set, (**C**–**D**) validation set and (**E**–**F**) pooled analysis. MST indicates median event-free survival times (in months). Patient numbers may not add up to 100% of available subjects because of missing genotyping data.

Supplementary Table 1: Selected demographic and clinical characteristics of gastric cancer patient population

Variables	Training set (*n* = 326) No. (%)	Validation set (*n* = 704) No. (%)	*P* value	Total (*n* = 1030) No. (%)
Sex				
Male	243 (74.5)	544 (77.3)		787 (76.4)
Female	83 (25.5)	160 (22.7)	0.337[a]	243 (33.6)
Tumor site				
Proximal	98 (30.1)	196 (27.8)		294 (28.5)
Body	104 (31.9)	257 (36.5)		361 (35.0)
Distal	124 (38.0)	251 (35.7)	0.353[a]	375 (36.5)
TNM stage				
I	56 (17.2)	147 (20.9)		203 (19.7)
II	143 (43.9)	339 (48.2)		482 (46.8)
III	93 (28.5)	163 (23.2)		256 (24.8)
IV	34 (10.4)	55 (7.7)	0.079[a]	89 (8.7)
Organ metastasis				
Liver	21 (61.8)	36 (65.5)		57 (64.0)
Coeliac	5 (14.7)	9 (16.4)		14 (15.7)
Other	8 (23.5)	10 (18.1)	0.828[a]	18 (20.3)
Differentiation				
Well	75 (23.0)	169 (24.0)		244 (23.7)
Moderate	93 (28.5)	185 (26.3)		278 (27.0)
Poor	152 (46.6)	336 (47.7)		488 (47.4)
Unknown	6 (1.9)	14 (2.0)	0.898[a]	20 (1.9)
Lauren classification				
Intestinal	147 (45.1)	293 (41.6)		440 (42.7)
Diffuse	170 (52.1)	391 (55.5)		561 (54.5)
Unknown	9 (2.8)	20 (2.9)	0.576[a]	29 (2.8)
ACT				
No	108 (33.1)	239 (33.9)		347 (33.7)
Yes	218 (66.9)	465 (66.1)	0.742[a]	683 (66.3)
Chemotherapy regimens[c]				
FOLFOX	211 (96.8)	454 (97.6)		665 (97.4)
CapeOX	7 (3.2)	11 (2.4)	0.609[a]	18 (2.6)
Relapse				
Yes	248 (76.1)	484 (68.8)		732 (71.1)
No	78 (23.9)	220 (31.2)	0.016[a]	298 (28.9)
Death				
Yes	208 (63.8)	390 (55.4)		598 (58.1)
No	118 (36.2)	314 (44.6)	0.011[a]	432 (41.9)
Age (years), median (range)	57 (21–81)	57 (20–83)	0.793[b]	57 (20–83)
Follow-up time (months), median (range)	76 (3–112)	58 (3–112)	<0.001[b]	62 (3–112)

Notes: TNM indicates tumor-node-metastasis; ACT, adjuvant chemotherapy.
[a]The *P* values were calculated using a Pearson Chi-Square test.
[b]The *P* values were calculated using a Mann-Whitney *U*-test.
[c]Only including gastric cancer patients receiving chemotherapy (*n* = 683).

Supplementary Table 2: Association of SNP rs6976500 with clinical outcomes of GC patients stratified by age, sex and tumor site

Variables	Genotypes	Deaths/Total[a] 597/1025	HR[b] (95% CI)	*P*	Relapses/Total[a] 729/1025	HR[b] (95% CI)	*P*
Age							
≤57	CC	150/301	Reference		186/301	Reference	
	CG + GG	132/202	1.51 (1.19–1.90)	0.001	166/202	1.78 (1.44–2.19)	<0.001
>57	CC	152/295	Reference		188/295	Reference	
	CG + GG	163/227	1.74 (1.39–2.18)	<0.001	189/227	1.69 (1.38–2.08)	<0.001
Sex							
Male	CC	252/475	Reference		309/475	Reference	
	CG + GG	213/308	1.57 (1.31–1.89)	<0.001	255/308	1.67 (1.42–1.98)	<0.001
Female	CC	50/121	Reference		65/121	Reference	
	CG + GG	82/121	1.97 (1.39–2.81)	<0.001	100/121	2.12 (1.55–2.91)	<0.001
Tumor site							
Proximal	CC	84/170	Reference		103/170	Reference	
	CG + GG	87/125	1.74 (1.29–2.36)	<0.001	102/125	1.85 (1.40–2.44)	<0.001
Body	CC	105/208	Reference		132/208	Reference	
	CG + GG	118/164	1.70 (1.31–2.22)	<0.001	134/164	1.72 (1.36–2.19)	<0.001
Distal	CC	113/218	Reference		139/218	Reference	
	CG + GG	90/140	1.45 (1.10–1.91)	0.009	116/140	1.69 (1.32–2.16)	<0.001

Note: HR, hazard ratio; CI, confidence interval.
[a] Numbers may not add up to 100% of available subjects because of missing genotyping data.
[b]Adjusted by age, sex, tumor site, differentiation, Lauren classification, TNM stage and adjuvant chemotherapy where appropriate.

Supplementary Table 3: Association of SNP rs6976500 with overall survival and recurrence-free survival of GC patients stratified by adjuvant chemotherapy (ACT)

SNP	In patients with ACT[a]							In patients without ACT[a]					
	Genotype	Deaths/ Total[b]	HR[c] (95% CI)	*P*	Recurrence/ Total[b]	HR[c] (95% CI)	*P*	Deaths/ Total[b]	HR[c] (95% CI)	*P*	Recurrence/ Total[b]	HR[c] (95% CI)	*P*
Training set													
rs6976500	CC	53/85	Reference		54/85	Reference		27/45	Reference		39/45	Reference	
	CG + GG	63/82	1.37 (1.01–1.95)	0.048	69/82	1.44 (1.03–1.96)	0.036	16/22	1.71 (1.15–3.10)	0.009	22/22	2.02 (1.18–3.49)	0.011
Validation set													
rs6976500	CC	129/249	Reference		163/249	Reference		25/39	Reference		30/39	Reference	
	CG + GG	91/159	1.15 (0.88–1.50)	0.309	123/159	1.28 (1.01–1.62)	0.039	43/53	1.46 (1.06–1.79)	0.018	43/53	1.49 (1.09–1.83)	0.002
Pooled analysis													
rs6976500	CC	182/334	Reference		52/82	Reference		217/334	Reference		69/82	Reference	
	CG + GG	154/241	1.34 (1.08–1.69)	0.008	59/75	1.45 (1.02–1.80)	0.043	192/241	2.17 (1.21–3.63)	<0.001	65/75	1.85 (1.13–2.95)	<0.001

Note: HR, hazard ratio; CI, confidence interval.
[a]Only including stage II and stage III GC patients received FOLFOX-based ACT.
[b]Numbers may not add up to 100% of available subjects because of missing genotyping data.
[c]Adjusted by age, sex, tumor site, differentiation, Lauren classification and TNM stage where appropriate.

Supplementary Table 4: 13 potential transcriptional factor binding sites near rs6976500 (−912 position) were predicted in sequence of SSBP1 5′-UTR (NT_007933.16)

Factor name	Start Position	End Position	Dissimilarity	Sequence	RE Equally	RE Query
GR-alpha	−927	−923	8.073878	GCAGG	5.94922	4.14484
	−908	−904	8.281568	CGAGG	5.94922	4.31353
TFII-I	−903	−898	9.512894	GTGTCC	5.57739	5.02460
STAT4	−934	−929	4.411765	GGAAGC	1.48730	1.26190
c-ETs-1	−936	−930	4.654478	CTGGAAG	0.65070	0.65440
Elk-1	−938	−930	10.962309	GCCTGGAAG	0.20334	0.15177
MyoD	−932	−923	4.956938	AAGCAGCAGG	0.00871	0.00526
AP-2alphaA	−927	−922	0.4638190	GCAGGC	0.74365	0.36788
	−899	−894	0.226186	CCAGGC	0.74365	0.36788
ENKTF-1	−927	−920	12.629773	GCAGGCCA	1.67322	1.27747
AR	−905	−897	5.644986	GGTGTGTCC	0.04067	0.02164
CAC-binding protein	−894	−887	2.152461	GCTCCACC	0.13943	0.06148
NF-1	−902	−893	6.908963	GTGTCCAGGC	0.07843	0.03701

Supplementary Table 5: Demographic and clinicopathological characteristics of the 70 GC patients for immunohistochemical assay

Variables	Number of patients. (%) $N = 70$
Age (years), median (range)	58 (28–81)
Sex	
Male	49 (70.0)
Female	21 (30.0)
Tumor site	
Proximal	19 (27.2)
Body	22 (31.4)
Distal	29 (41.4)
TNM stage	
I	14 (20.0)
II	27 (38.6)
III	23 (32.9)
IV	6 (8.5)
Differentiation	
Well	9 (12.9)
Moderate	29 (41.4)
Poor	31 (44.3)
Unknown	1 (1.4)
Lauren classification	
Intestinal	29 (41.4)
Diffuse	36 (51.4)
Unknown	5 (7.6)

Note: GC, gastric cancer; TNM, Tumor-Node-Metastasis.
